# The role of MBD2 in immune cell development, function, and autoimmune diseases

**DOI:** 10.1038/s41420-025-02563-0

**Published:** 2025-06-19

**Authors:** Yunfei Zhang, Yufeng Fan, Ying Hu, Xiaocui Wang, Bin Wen, Xuemei Duan, Haonan Li, Shumin Dong, Ze Yan, Weiwei Zhang, Yukai Jing

**Affiliations:** 1 Department of Clinical Laboratory, Third Hospital of Shanxi Medical University, Shanxi Bethune Hospital, Shanxi Academy of Medical Sciences, Tongji Shanxi Hospital, Taiyuan, China; 2 Department of Anesthesiology, Third Hospital of Shanxi Medical University, Shanxi Bethune Hospital, Shanxi Academy of Medical Sciences, Tongji Shanxi Hospital, Taiyuan, China

**Keywords:** Autoimmunity, Autoimmune diseases

## Abstract

DNA methylation is a key epigenetic modification that regulates gene expression, cell differentiation, and genome stability. Aberrant DNA methylation patterns, including the hypermethylation or global hypomethylation of tumor suppressor genes, are strongly associated with various human diseases, such as cancer, autoimmune disorders, and metabolic syndrome. DNA methylation predominantly occurs at CpG dinucleotides, influencing transcription by altering chromatin structure and accessibility. MBD2 (Methyl-CpG-binding proteins 2) play a crucial role in interpreting these epigenetic marks and regulating downstream gene expression. In disease contexts, aberrant DNA methylation disrupts cellular homeostasis by silencing key regulatory genes or activating pathological pathways. Current research primarily focuses on MBD2 in cancer, with less emphasis on its role in autoimmune diseases. This review discusses the role of MBD2 in regulating immune cell development and differentiation through epigenetic mechanisms, particularly DNA methylation and its regulatory components. Furthermore, it highlights the mechanistic contributions of MBD2 to autoimmune diseases such as systemic lupus erythematosus and evaluates its potential as a novel therapeutic target for these conditions.

## Facts

- Heterogeneity in MBD2 regulation: gene expression patterns of MBD2 exhibit disease-specific variations across autoimmune disorders, accompanied by distinct regulatory mechanisms in different pathological contexts.
- Prevalence of DNA hypomethylation: a hallmark of most autoimmune diseases is the widespread predominance of DNA hypomethylation, suggesting a shared epigenetic disruption in immune cell homeostasis.
- Therapeutic potential of MBD2 targeting: emerging therapies focusing on DNA methylation modulation have shown preliminary success, underscoring MBD2 as a mechanistically rational and clinically actionable target for autoimmune disease management.

## Introduction

DNA methylation, a vital epigenetic mechanism, regulates immune cell development and function by influencing gene expression, chromatin remodeling, and genomic stability. This modification, predominantly occurring at CpG dinucleotides, mediates gene silencing by recruiting methyl-CpG-binding proteins such as MBD2 and histone-modifying complexes, thereby shaping immune responses [1]. For instance, the differentiation of T cells and B cells is intricately governed by DNA methylation patterns that ensure the activation or repression of lineage-specific genes. Disruptions in these patterns can skew immune cell differentiation, leading to impaired responses or hyperactivation [2]. MBD2, as a key reader of methylated DNA, further modulates chromatin accessibility and transcriptional activity in immune cells, underscoring the crucial role of methylation in maintaining immune homeostasis. MBD2 primarily exerts its effects via the NURD (nucleosome remodeling and histone deacetylase) complex, modulating chromatin accessibility and transcriptional activity [3]. Dysregulated MBD2 expression has been linked to various pathological conditions, including autoimmune diseases and cancers [4, 5].

DNA methylation contributes to the pathogenesis of various diseases, including autoimmune disorders, cancers and chronic inflammation, where aberrant methylation patterns represent characteristic features. Research highlights that SLE (systemic lupus erythematosus), Sjögren’s syndrome [6], and multiple sclerosis [7] exhibit hypomethylation in immune cells, contributing to dysregulated immune responses and chronic inflammation [5]. MBD2-mediated DNA methylation regulation has spurred development of DNMT and MBD protein inhibitors, which correct aberrant gene expression and attenuate disease progression through epigenetic remodeling, including inhibitors of DNMTs (DNA methyltransferases) [8] and MBD (methyl-CpG binding domain) proteins [9], have shown promise in restoring normal gene expression and mitigating disease progression [10]. These findings emphasize the central role of DNA methylation in bridging epigenetic regulation and disease pathology. However, the specific mechanisms by which MBD2, an important component of DNA methylation, contribute to different autoimmune diseases and its role in various immune cells remain unclear.

This study systematically summaries the dual role of MBD2 in orchestrating DNA methylation-dependent gene silencing through its recruitment of the NuRD complex to methylated CpG sites. This study provides a mechanistic overview of MBD2’s regulatory functions in both innate and adaptive immune cells, with specific emphasis on its contributions to autoimmune pathogenesis. Therapeutic targeting of MBD2 demonstrates potential to correct epigenetic dysregulation in autoimmunity, with preclinical studies identifying tractable strategies for clinical translation.

## Structure and activation mechanism of MBD2

### The structure and function of MBD2

DNA methylation is a key epigenetic modification that is essential for mammalian survival and development. Studies have shown that DNA methylation plays a critical role in cell development, differentiation, and transcriptional regulation. Currently, DNA mCG (methylation at CpG dinucleotides) is associated with stable gene repression, this modification directly influences transcription by altering the way and position in which protein factors bind to DNA [1]. Methylated promoters may repel transcription factors, leading to gene silencing, though certain transcription factors can also recognize specific methylated sequences associated with active transcription. In addition, DNA methylation can indirectly regulate transcription through the binding of specific proteins, referred to as “readers” of CpG methylation [11].

MBD family proteins typically act as mediators, linking DNA methylation (primarily in the CpG context) with other chromatin and histone modification complexes. The MBD protein family comprises MeCP2 and MBD1-6, with each member exhibiting unique structural domains and functional specialization. These proteins share a conserved MBD domain that binds methylated DNA, but their divergent C-terminal domains - including TRD (transcriptional repression domains), CXXC zinc fingers and enzymatic motifs - enable distinct roles in chromatin remodeling, DNA repair, and transcriptional regulation. For instance, MBD2 and MBD3 are mutually exclusive components of the NuRD complex, while MBD4 integrates DNA methylation sensing with glycosylase activity to repair mismatched bases. Among them, MBD2 is a widely expressed and highly conserved protein that interacts with the NuRD complex. MBD2 is encoded by six coding exons and one non-coding exon and exists in three isoforms: MBD2a, MBD2b, and MBD2c [12]. Current research suggests that MBD2 suppresses gene expression by recruiting the NuRD complex [13, 14]. Notably, certain protein isoforms, including MBD2a, have been shown to mediate transcriptional reactivation of silenced genes [15, 16], suggesting a potential link to TACC3 expression regulation. This functional association may provide critical insights into the mechanistic underpinnings of MBD2’s biological activities, particularly its dual role in epigenetic modulation.

### Ligands bound and the mechanism

MBD2 is a key component of the NuRD complex, which consists of ATP-dependent remodeling enzymes, histone deacetylases, and DNA-binding proteins, facilitating both nucleosome remodeling and transcriptional regulation [17]. Initially, MBD2 was thought to solely recruit NuRD to methylated genomic regions, promoting histone deacetylation and chromatin compaction, thereby repressing transcription [18]. However, emerging evidence reveals a more nuanced role for MBD2, which localizes to actively transcribed, unmethylated genomic loci. This observation suggests that the NuRD complex may direct MBD2 to these regions rather than solely operating through methylation-dependent recruitment mechanisms [16]. The functional divergence of MBD2 and MBD3 within the NuRD complex is evidenced by their structural specialization in DNA binding and distinct phenotypic outcomes upon knockout. MBD2 preferentially incorporates into NuRD complexes targeting methylated CpG islands, while MBD3 binds both methylated and non-methylated DNA with lower selectivity [19]. This complexity emphasizes the need to further explore MBD2’s dynamic role in transcriptional regulation, both as part of the NuRD complex and independently, to clarify its target gene interactions and regulatory mechanisms.

MBD2, while primarily associated with the NuRD corepressor complex, also interacts with various protein complexes that may influence both its binding to NuRD and its independent functions. Notably, post-translational methylation by PRMT1 and PRMT5 modifies the N-terminal RG-rich region of MBD2a, reducing its affinity for the NuRD complex and methylated DNA, which is a unique regulatory mechanism not seen with MBD3 [20]. Furthermore, MBD2 has been implicated in transcriptional activation through its interactions with TACC3 and the histone acetyltransferase pCAF, as well as RNA helicase A [15], suggesting roles beyond repression. These findings indicate that MBD2’s interactions could either mediate NuRD-dependent activities or represent distinct functions.

### Important functional regions for mCPG binding

**Fig. 1:**
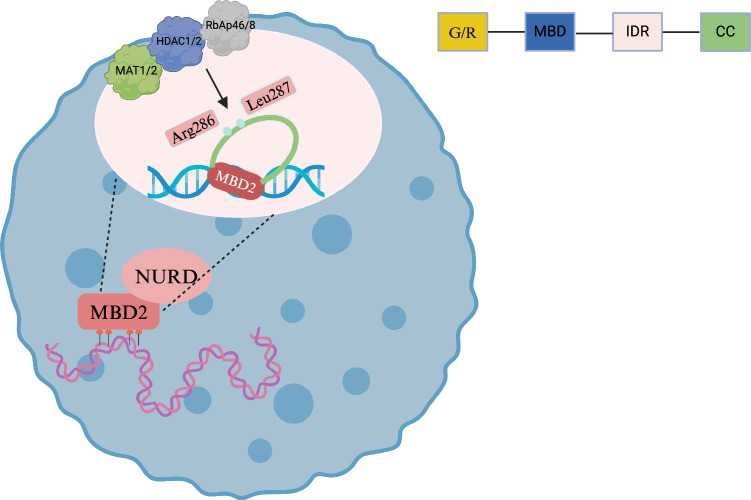
Specialized sites within the intrinsically disordered region (IDR) of the MBD2 structure that bind NuRD complexes. MBD2 is composed of four domains: an N‑terminal glycine–arginine (GR) repeat region, a methyl‑CpG binding domain (MBD), an intrinsically disordered region (IDR), and a C‑terminal coiled‑coil helix. The C‑terminal helix is involved in recruitment of CHD4 and gene silencing through interaction with the NuRD subunit p66. The IDR significantly enhances DNA‑binding affinity and mediates recruitment of NuRD components RbAp48, HDAC2, and MTA2 via residues Arg286 and Leu287. Mutation of these two residues abrogates histone interactions. The figure was created on the Biorender (https://www.biorender.com/).

The MBD2 gene produces three alternatively spliced isoforms that exhibit distinct functional properties through domain-specific truncations: full-length MBD2a containing all four domains, MBD2b lacking N-terminal GR (glycine-arginine) repeats critical for HDAC1 interaction, and MBD2c (historically termed MBD2t) missing the C-terminal CC (coiled-coil) domain required for NuRD complex incorporation [21]. In a previous study, the C-terminal convoluted helix of MBD2 interacts with the p66 component of NuRD, facilitating CHD4 recruitment and gene silencing. The IDR markedly enhances the protein’s binding affinity for methylated DNA [22]. Furthermore, the IDR recruits the histone deacetylase core components of NuRD, including RbAp48, HDAC2, and MTA2, through a critical contact region that requires two consecutive amino acid residues, Arg286 and Leu287 (Fig. 1). Mutating these residues disrupts MBD2’s interaction with the histone deacetylase core. The GR region domain critically modulates MBD2’s mCpG binding affinity and mediates its incorporation into the NuRD complex. The MBD2b isoform lacking GR repeats fails to rescue neuronal differentiation defects in MBD3-knockout ESCs (embryonic stem cells), indicating GR-dependent functional compensation between these paralogs during neurogenesis. These findings enhance our understanding of the multidimensional interactions between MBD2 and the NuRD complex, shedding light on its functional regulatory mechanisms.

## Role of MBD2 in regulating immune cell function and mechanisms

### Adaptive immune cells

#### CD4^+^ T cells

**Fig. 2:**
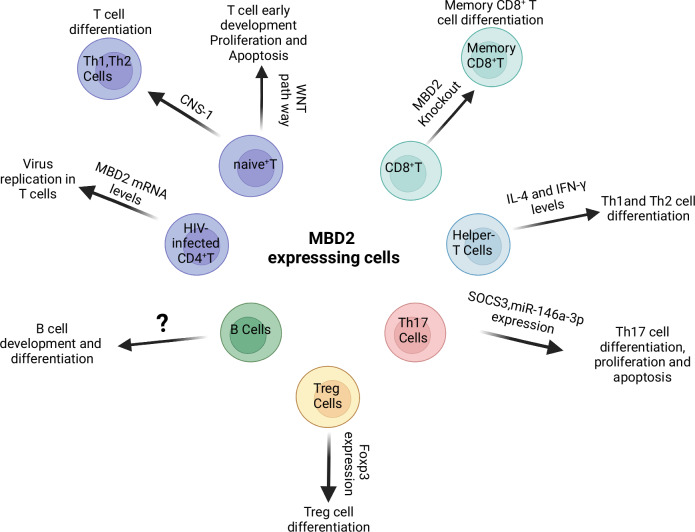
The role of MBD2 in adaptive immune cells. In various adaptive immune cell types, MBD2 influences cellular development and differentiation at multiple levels—transcriptional regulation, signal transduction, and post-transcriptional control—via diverse mechanisms such as binding to the conserved non‑coding sequence CNS-1, modulating expression of signaling proteins (SOCS3 and Foxp3), regulating cytokine secretion (IL-4 and IFN-γ), and controlling its own expression. Key cellular subsets include Th1 (T helper 1 cells), Th2 (T helper2 cells), CD8^+^T lymphocytes (cytotoxic T cells), and memory CD8^+^T cells (antigen-experienced cytotoxic memory cells). The figure was created on the Biorender (https://www.biorender.com/).

MBD2 influences cell development and differentiation by recruiting the NuRD complex and thus regulating DNA methylation (Fig. 2). Mi Zhou et al. demonstrated that MBD2 regulates the early stages of T cell development, particularly in DN (double-negative) T cells within the thymus, through the WNT signaling pathway. This regulation specifically affects the apoptosis and proliferation of DN cells [23]. The present findings indicate that MBD2 plays a crucial regulatory role in T cell development and differentiation.

Moreover, MBD2 has also been shown to be essential for CD4^+^ T cell proliferation. In CD4^+^ T cells treated with exosomes, microRNA exerts glycolytic effects by targeting MBD2, thereby enhancing CD4^+^ T cell proliferation [24]. MBD2 may regulate mitochondrial function and bioenergetic metabolism during T cell development through OPA1-mediated mitochondrial fusion [25]. This underscores the dual role of MBD2 in modulating both CD4^+^ T cell differentiation and metabolic processes.

Paradoxically, in HIV-infected cells, MBD2-mediated silencing of the viral LTR through NuRD-dependent deacetylation establishes latency, but its inactivation risks unleashing both viral reactivation and host immune exhaustion [26], this context-dependent duality underscores the need for precision targeting of MBD2’s functional modules in immunotherapy. This suggests that MBD2 may ultimately contribute to DNA hypomethylation and aberrant gene expression in peripheral T cells of patients with autoimmune diseases by modulating DNA methylation levels and patterns, and inhibiting gene transcription (Table 1).

**Table 1:** Role of MBD2 in regulating immune cell function and mechanisms of activation.

Cells	MBD2 binding sites	MBD2 inactivation method	MBD2 inactivation phenotype	Outcomes	Ref
CD4^+^T Cells	CNS-1	MBD2^−/−^	Reduced expression of IL-4, IL-5, and IL-13	Affect the transformation of naïve T cells into TH 1 and TH 2 cells; promoting the transformation of T cells into inducible NK cells	[23, 24]
CD8^+^T Cells	NO	MBD2^−/−^virus-infected		Restricting the differentiation of naïve CD8 ^+^ T cells into memory CD8 ^+^ T cells	[29, 30]
Tfh, Th17 Cells	SOCS3; miR-146a-3p	MBD2^−/−^	IL-4 and IFN-γ were elevated	Restriction of Tfh cells, and the development of Th 17 cells	[40, 41]
Treg Cells	Foxp3	MBD2^−/−^	The Treg-specific demethylation region (TSDR) demethylation	Maintain hypermethylation of the Treg-specific demethylation regions (TSDR)	[42]
Macrophage	SHIP	MBD2^−/−^	TGF-β 1, and the PDGF was decreased	Inhibition of the PI3K/Akt pathway to alleviate M2 macrophage-mediated both diseases	[48, 49]
Dendritic cell	TRAF6	MBD2^-/-^	NO	DCs have impaired basic functions, such as antigen uptake, processing, and presentation capacity	[51, 54]

#### CD8^+^ T cells

Memory CD8^+^ T cells play a pivotal role in the speed of viral infection response. These cells develop from a precursor population during the effector immune response [27, 28]. In MBD2-deficient virus-infected mice, MBD2 played a critical role in the differentiation of naïve CD8^+^ T cells into effector and memory cells, with a notable defect in memory CD8^+^ T cell differentiation. Furthermore, the adaptability of MBD2-associated resident memory CD8^+^ T cells across different tissues are not entirely consistent [29]. The delay in memory CD8^+^ T cell emergence may be linked to functional redundancy between MBD2 and MBD3 [30]. These findings underscore the involvement of MBD2 in regulating immune cell proliferation, differentiation, and exhaustion processes, where it influences immune cell states through DNA methylation and interactions with other immune-modulatory factors.

Since MBD2 acts as a co-repressor within the Mi-2/NuRD complex, MBD2 deletion alone may be insufficient to fully reactivate Mi-2/NuRD target gene expression [31, 32]. This further implies that the role of MBD2 within the NuRD complex varies across different immune cells.

#### Th1, Th2 cells

It has been demonstrated that the differentiation of Th cells relies on proper gene methylation, as evidenced by experiments utilizing methylation inhibitors [33]. In CD4^+^ T cells, MBD2 further affects the ability of TH2 cells to secrete IL-4 and IL-13 by affecting the ability to bind to CNS-1 (conserved noncoding sequences), which ultimately affects the transformation of naïve T cells into TH1 and TH2 cells [34]. T cell subsets isolated from MBD2-deficient mice demonstrate significantly enhanced cytokine production compared to wild-type counterparts, implicating MBD2 as a negative regulator of inflammatory signaling in adaptive immunity [35]. This suggests that MBD2 regulates the fine-tuned differentiation of CD4^+^ T cells by modulating DNA methylation, interacting with target genes such as CNS-1, and influencing the secretion of key cytokines like IL-4.

In response to microbial invaders, progenitor cells (naïve helper T cells) undergo proliferation and maturation, leading to the secretion of high levels of either Th1 or Th2 cytokines. Th1 cells secrete IFN-γ, while Th2 cells secrete IL-4 [36, 37]. Studies have shown that MBD2 restricts the induction of cytokine genes during helper T-cell differentiation. Furthermore, MBD2 has been found to mediate IL-4 silencing and compete with Gata-3 for genetic regulation [38]. This suggests that during T cell differentiation, MBD2 influences the differentiation of Th1 and Th2 cells through its role in DNA methylation and gene silencing, thereby regulating the cytokine secretion profiles. Such precise epigenetic regulation facilitates the functional responses of immune cells during specific immune reactions.

#### Th17 cells

In severe asthma, Th17 cells were abnormally elevated, with overexpression or silencing of the MBD2 gene leading to corresponding changes in Th17 cell numbers [39]. Further studies revealed that MBD2 can downregulate SOCS3 expression, thereby affecting Th17 cell differentiation [40]. Additionally, MBD2 influenced the overexpression of miR-146a-3p, which inhibited the in *vivo* response of Th17 cells and alleviated severe asthma [41]. These findings strongly support the role of MBD2 in regulating Th17 cell differentiation and further modulating disease.

#### Treg cells

It has been demonstrated that MBD2 can bind to Foxp3, which is a unique and evolutionarily conserved CpG-rich island found within the non-intronic upstream enhancer regions of Foxp3. Specific sites of Foxp3 were unmethylated in natural Treg (nTreg) and highly methylated in naïve CD4^+^ T cells, activated CD4^+^ T cells, and peripheral TGF-β-induced Tregs [42]. This finding indicates that the nTreg and TGF-β-induced Treg enhancers have distinct structural characteristics. Additionally, it suggests that MBD2 functions differently based on the specific location of the Foxp3 enhancer. Furthermore, deletion of MBD2 in Tregs led to demethylation of the TSDR (Treg-specific demethylation region) and a reduction in the inhibitory function of Tregs both in *vivo* and in *vitro* [43, 44]. These results suggest that MBD2 plays a crucial role in Treg cell differentiation.

#### B cell

The Mi-2/nucleosome remodeling and deacetylase (NuRD) chromatin remodeling complex is a key regulator of chromatin structure and DNA accessibility [45, 46]. Research has shown that MBD2 not only participates in transcriptional repression through direct interaction with CHD4 but also regulates chromatin structure and the recruitment of transcription factors via its interaction with the Mi-2/NuRD complex [47]. These findings suggest that MBD2 may play a key role in B cell development and peripheral differentiation; however, the precise mechanisms remain unclear.

### Innate immune cells

#### Macrophage

**Fig. 3:**
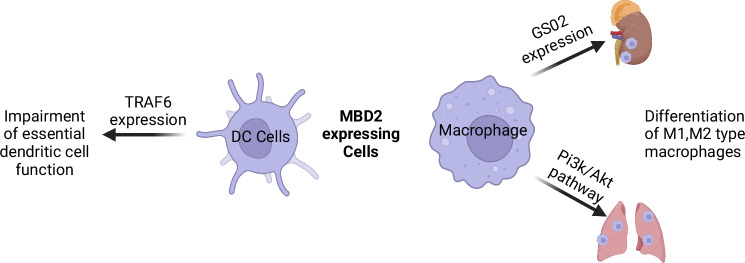
The role of MBD2 in innate immune cells. In innate immune cells, MBD2 modulates dendritic cell function by regulating TRAF6 expression and influences macrophage differentiation through GS02 expression and activation of the PI3K signaling pathway. The figure was created on the Biorender (https://www.biorender.com/).

Macrophages can polarize into M1 or M2 types depending on the environmental context, with M2 macrophages playing key roles in various diseases. The attenuation of renal fibrosis in mice subjected to UUO and I/R was first demonstrated upon knockdown or silencing of MBD2 in macrophages, an effect attributed to its binding to GS02 (Fig. 3). These shifts in macrophage polarization contribute to increased renal fibrosis, likely through induced hypomethylation of target gene promoters [48].

In allergic asthma, M2 macrophages secrete inflammatory factors such as IL-13, CCL17, CCL22, and eosinophil chemotactic factors. The absence of MBD2 in macrophages significantly reduced the M2-biased macrophage phenotype, suggesting that MBD2 may also regulate macrophage M2 programs in an asthmatic setting, consistent with previous findings in renal fibrosis [49]. However, the specific role of MBD2 in macrophage polarization during asthma progression remains poorly understood. In pulmonary fibrosis, M2 macrophages release TGF-β1 (Transforming growth factor-β1) and PDGF (Platelet-derived growth factor), which promote fibroblast differentiation into myofibroblasts, initiating pulmonary fibrosis [49, 50]. Interestingly, MBD2 deficiency mitigates M2 macrophage-mediated pathology in both diseases by inhibiting the PI3K/Akt pathway.

#### Dendritic cell

**Fig. 4:**
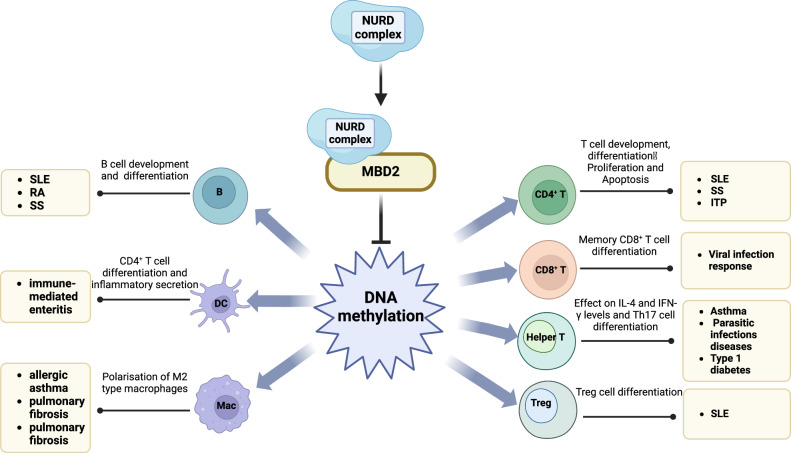
Role of MBD2 in regulating immune cell function and mechanisms. Given MBD2’s roles in DNA methylation and in both adaptive and innate immune cells, it contributes to the pathogenesis of various autoimmune diseases. MBD2 recognizes methylated CpG sites via its methyl-CpG binding domain and, in concert with the NuRD chromatin-remodeling complex, modulates the epigenetic programming of adaptive and innate immune cells. The figure was created on the Biorender (https://www.biorender.com/).

DCs (Dendritic cells) induce the differentiation of CD4^+^ T cells into Th cells, particularly Th2 cells. MBD2 deficiency impairs fundamental dendritic cell functions, including antigen uptake, processing, and presentation, as well as CD4^+^ T cell activation, which is inextricably linked to the fact that MBD2 affects levels of TNF-α, IL-6 and cytokines [51]. Furthermore, MBD2 regulates the DNA methylation status of specific genes on dendritic cells, such as Foxp3 and IL-10, and influences the interaction with polycomb repressive complexes (PRC1/PRC2) to epigenetically modulate Hox genes, contributing to the maintenance of intestinal epithelial stability [52, 53]. In the context of microbiota-mediated immune tolerance, MBD2 modulates dendritic cell immune responses by influencing TRAF6 (TNF receptor-associated factor 6) expression, particularly in dendritic cells. Additionally, MBD2 alters the gene expression of key immune modulators such as IL-10 and critical pro-inflammatory cytokines including IL-12 and TNF-α, thereby modulating dendritic cell function [54]. And mbd2-deficient mice exhibit chronic intestinal inflammation following a single mucosal injury, with MBD2-deficient intestinal T cells overexpressing IFN-γ in experimental colitis [55]. Additionally, MBD2 deficiency exacerbates DSS-induced colitis by limiting the regulatory capacity of CD11c^+^ dendritic cells and colonic ECs (Epithelial cells) [56]. By modulating key molecules such as IL-4, IL-10, TNF-α, IL-6, and Foxp3, MBD2 helps regulate immune homeostasis, preventing excessive immune responses or immune evasion. The epigenetic regulatory role of MBD2 is crucial in diseases such as intestinal immune homeostasis, allergic responses, immune tolerance, and intestinal cancers (Fig. 4).

## The role of MBD2 in autoimmune diseases

### Systemic lupus erythematosus

In various autoimmune diseases, CD4^+^ T cells from patients with lupus exhibit a hypomethylation profile. Specifically, active lupus patients show lower methylation levels compared to inactive patients, while MBD2 expression is significantly higher in lupus patients [57]. In SLE patients, genomic methylation indices inversely associate with MBD2 expression levels. Disease activity (quantified by SLEDAI) further demonstrates a negative correlation with methylation status, while exhibiting a positive correlation with MBD2 mRNA abundance, suggesting coordinated dysregulation of epigenetic machinery in SLE pathogenesis [58]. These results suggest that elevated MBD2 significantly impacts the hypomethylation of CD4^+^ T cells and the progression of lupus [57, 59]. Mechanistically, MBD2 cooperates with DNMT1 through physical interaction to orchestrate methylation homeostasis—where MBD2 recognizes methylated CpG sites while DNMT1 maintains methylation patterns during DNA replication [60]. Furthermore, there is a close association between the methylation transferase DNMT and MBD2, particularly DNMT1.

**Fig. 5:**
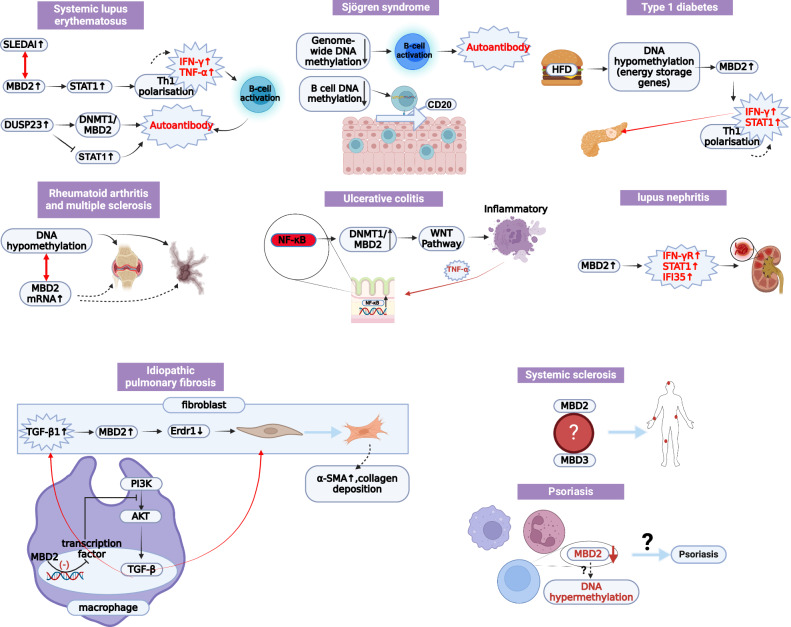
The role of MBD2 in autoimmune diseases. Systemic lupus erythematosus (SLE): Elevated MBD2 induces hypomethylation in CD4^+^T‑cells, activates STAT1‑driven Th1 polarization with increased IFN‑γ and TNF‑α production, and amplifies inflammation via a positive feedback loop; Sjögren’s syndrome (SS): Global DNA hypomethylation promotes aberrant B‑cell activation and salivary‑gland infiltration, leading to epithelial–immune interactions, anti‑SSA/SSB antibody production, glandular damage, and xerostomia; Type 1 diabetes (T1D): High‑fat diet–induced hypomethylation of metabolic genes upregulates MBD2, enhances STAT1‑mediated Th1 polarization (IFN‑γ, TNF‑α), and directly damages pancreatic β‑cells; MBD2 deficiency causes uncontrolled STAT1 activity and disease exacerbation; Ulcerative colitis (UC): Inflammation‑triggered NF‑κB nuclear translocation upregulates DNMT1 and MBD2, resulting in hypermethylation of WNT‑pathway tumor suppressors such as APC, establishing a TNF‑α/NF‑κB vicious cycle and persistent mucosal injury; Idiopathic pulmonary fibrosis (IPF): TGF‑β1 activates MBD2, represses Erdr1, and induces fibroblast‑to‑myofibroblast transdifferentiation, while inhibiting M2 macrophage polarization to create a pro‑fibrotic microenvironment; Psoriasis: In contrast to other diseases, global DNA hypermethylation coincides with reduced MBD2 expression, the mechanism remains unclear; Systemic sclerosis (SSc): MBD3 competes with MBD2 for the NuRD complex to regulate epigenetic balance. The mechanism is not clear. All figures were created on the Biorender (https://www.biorender.com/).

Patients with systemic lupus erythematosus exhibit significantly reduced DNA methylation compared to healthy controls. Additionally, mRNA levels of MBD2 and DNMT1 were significantly elevated in SLE patients compared to those in controls, with a positive correlation observed between DNMT1 and MBD2 mRNA expression. Furthermore, MBD2 mRNA levels increased with disease severity [61]. DUSP23(Dual-specificity protein phosphatase 23), located on human chromosome 1, is identified as a susceptibility gene for systemic lupus erythematosus. In SLE patients, DUSP23 mRNA levels are significantly elevated and positively correlated with transcript levels of DNA methylation-related enzymes, including DNMT1, DNMT3A, DNMT3B, MBD2, and MBD4 [62]. DUSP3, DUSP22, and VH1 regulate IFN (interferon) and interleukin signaling via STAT protein dephosphorylation, contributing to autoreactive T-cell activation and immune tolerance loss in SLE [63, 64]. Notably, increased IFN-α activity is frequently detected in the serum of SLE patients [65–67]. The overexpression of MBD2 may facilitate gene silencing by binding to hypomethylated DNA regions and recruiting DNMTs or histone-modifying enzymes. The positive correlation between DUSP23 and DNMTs/MBDs suggests that DUSP23 may amplify autoimmune gene expression through epigenetic regulation, while simultaneously suppressing negative feedback signals via STAT dephosphorylation, thereby establishing a self-reinforcing “epigenetic-signaling” vicious cycle (Fig. 5). However, while current studies primarily focus on clinical statistics, the mechanisms by which MBD2 contributes to the pathogenesis of lupus remain insufficiently explored.

### Sjögren syndrome

A genome-wide methylation assay of European women with SS (Sjögren’s syndrome) revealed global hypomethylation of genes in SS patients [68]. Furthermore, studies of DNA methylation profiles in naïve CD4^+^ T cells, B cells, and salivary gland epithelial cells provided additional evidence of aberrant DNA methylation in SS patients [69, 70]. Abnormally activated B cells play a key role in various autoimmune diseases. In SS, activated B cells accumulate around salivary gland epithelial cells, and the extent of demethylation in these cells strongly correlates with B cell infiltration [71]. In conclusion, DNA hypomethylation may act as a molecular bridge linking epithelial cell dysfunction and B cell hyperactivity, creating a self-amplifying inflammatory loop.

### Type 1 diabetes

A high-fat diet (HFD) has long been recognized as a potent environmental factor that induces obesity and insulin resistance [72]. Short-term high-fat diets are proposed to perturb skeletal muscle DNA methylation, with observed hypomethylation at loci linked to T1D (Type 1 diabetes), while aberrant methylation signatures may drive autoimmune activation in T1D pathogenesis, this is partly because HFD induces DNA demethylation of genes involved in energy storage, a process that is effectively inhibited by MBD2 knockdown [73]. Furthermore, MBD2 plays a critical role in regulating glucose homeostasis and lipid metabolism, which are closely linked to the development of HFD-induced obesity and insulin resistance [74]. Studies have shown that MBD2-deficient mice are more likely to develop symptoms of T1D, with the polarization of Th1 cells and the associated regulatory gene STAT1 being significantly upregulated during the disease process [75]. Additionally, the levels of cytokines IFN-γ, GM-CSF, and TNF-α secreted by Th1 cells are significantly elevated. MBD2 maintains the homeostasis of the Th1 program by binding to methylated CpG DNA within the Stat1 promoter, thereby preventing autoimmunity [75]. MBD2 emerges as a critical node linking HFD-induced metabolic stress, DNA methylation dysregulation, and Th1-driven autoimmune activation in T1D.

### Rheumatoid arthritis and multiple sclerosis

In rheumatoid arthritis and multiple sclerosis, patients exhibit global DNA hypomethylation compared to healthy individuals, accompanied by elevated mRNA expression levels of MBD2 and DNMT1. Published microarray data on multiple sclerosis indicate lower levels of the demethylase TET3 [76]. Furthermore, genes in the DNMT and MBD families are positively correlated with DNA methylation in various immune-related diseases [57]. These findings highlight the crucial role of DNA methylation in the pathogenesis of rheumatoid arthritis and multiple sclerosis.

### Ulcerative colitis

The hallmark of UC (ulcerative colitis) is chronic inflammation of the colon [77], characterized by significantly enhanced NF-κB activity in colonic epithelial cells and immune cells, which leads to elevated levels of pro-inflammatory cytokines. This cascade triggers an upregulation of DNMTs, which suppress tumour suppressor genes via promoter methylation [78]. Yi-Wen Huang and colleagues demonstrated that inhibiting DNA methylation at the promoter regions of the WNT pathway effectively reduced macrophage accumulation and improved disease outcomes by mitigating the activation of inflammatory signaling pathways, including NF-κB, particularly in UC [79]. NF-κB activation upregulates DNMT1/3A, which may collaborate with MBD2 to mediate aberrant methylation of pro-inflammatory genes, further amplifying inflammation.

### lupus nephritis

Abnormal activation of inflammatory factor signaling pathways plays an important role in lupus nephritis. IFN-γ is abnormally elevated in lupus nephritis and is associated with disease activity. Additionally, IFN-γR, STAT1, and IFI35—key components of the IFN-γ signaling pathway—were found to be significantly elevated and hypomethylated in LN kidney tissues, all of which are regulated by MBD2 [80]. The present findings suggest that MBD2 regulates inflammatory pathways through DNA methylation, thereby mitigating the severity of inflammation-driven disorders.

### Idiopathic pulmonary fibrosis

IPF (Idiopathic pulmonary fibrosis) is a progressive and fatal disease of lung interstitial fibrosis for which effective clinical treatments are lacking. Multiple studies by Congyi Wang have shown that MBD2 levels are elevated in both IPF patients and bleomycin-induced pulmonary fibrosis model mice, particularly in the myofibroblasts within fibrotic lungs [49]. Deletion of MBD2 in fibroblasts or myofibroblasts can protect against lung fibrosis in BLM-treated mice by inhibiting fibroblast differentiation into myofibroblasts. Mechanistically, TGF-β1 induces global DNA hypermethylation and MBD2 overexpression in lung fibroblasts. MBD2 selectively binds to methylated CpG sites within the Erdr1 promoter without altering its overall methylation level, repressing Erdr1 expression and promoting fibroblast differentiation into myofibroblasts, thus exacerbating IPF progression [81]. Furthermore, MBD2 inhibits M2-type macrophage polarization in the lung interstitial tissue, preventing further fibroblast-to-myofibroblast differentiation [49].

The dichotomous methylation patterns of Erdr1 (hypomethylation versus hypermethylation), though superficially paradoxical, exemplify the spatiotemporal complexity of epigenetic regulation in disease etiology, where site-specific DNA methylation exerts bidirectional control over pathological processes. In pulmonary fibrosis, DNA methylation exerts its pathological effects through cell-type-specific methylation dynamics—specifically, hypomethylation of pro-fibrotic genes and hypermethylation of anti-fibrotic genes in critical cellular subsets such as macrophages and fibroblasts—rather than through alterations in global methylation patterns of lung tissue or bulk cellular DNA [81]. MBD2 drives IPF progression through dual cell-type-specific mechanisms: epigenetic repression of the anti-fibrotic factor *Erdr1* in pulmonary fibroblasts, coupled with impairment of pro-resolving M2 macrophage function. This functional duality enables context-specific epigenetic regulation within discrete pulmonary niches, revealing its compartmentalized regulatory versatility (Table 2).

**Table 2:** The role of MBD2 in autoimmune diseases.

Disease	MBD2 ligands	Cells	MBD2 inactivation method	MBD2 inactivation phenotype	Contributing pathways	Ref
Systemic lupus erythematosus	DNMT1	CD4 T cells	NO	NO	MBD 2 ultimately causes hypomethylation of the relevant genes by affecting DNMT 1	[61, 62]
Sjögren syndrome	NO	B cells	NO	NO	MBD 2 affects B cell demethylation around the salivary glands	[70, 71]
Type 1 diabetes	STAT1	Th1 cells	MBD2^−/−^	The IFN- γ, GM-CSF, and TN-F- α levels increased significantly	MBD 2 maintains the homeostasis of the Th 1 program by binding to the methylated CpG DNA within the Stat 1 promoter	[73, 75]
Rheumatoid arthritis and multiple sclerosis	NO	NO	NO	NO	Patients presented global DNA hypomethylation compared to healthy individuals, along with elevated mRNA expression levels of MBD 2 and DNMT 1	[76]
Ulcerative colitis and lupus nephritis	IFN-γR, STAT1, and IFI35	Macrophage	MBD2^−/−^	IFN- γ, the activation of the NF- κ B-related pathway	MBD 2 controls the expression of molecules of inflammation-related pathways such as IFN- γ R, STAT 1, and IFI 35 and NF- κ B	[79, 80]
Idiopathic pulmonary fibrosis	Erdr1	Macrophage	MBD2^−/−^	Prevent fibroblast differentiation into myofibroblasts	MBD 2 selectively binds to the methylated CpG sites within the Erdr 1 promoter	[81]

### Systemic sclerosis

In SSc (systemic sclerosis), the expression of other MBD family molecules, such as the mRNA of MBD3 and MBD4, is reduced compared to controls, and a positive correlation exists between the relative levels of MBD4 and DNA methylation in the SSc group. Reduced expression of MBD3 and MBD4 reduces the methylation of autoimmune-related genes, leading to increased expression of these genes in lymphocytes, which contributes to the development of SSc. Conversely, MBD3 can counteract the effects of MBD2 by interacting with components of the nucleosome remodeling and deacetylase (NuRD) complex [57]. The specific role of MBD2 in systemic sclerosis requires further investigation.

### Psoriasis

Aberrant DNA methylation underlies cutaneous pathologies such as autoimmune dermatoses – exemplified by psoriasis and lupus erythematosus – and malignancies, with melanoma and squamous cell carcinoma representing characteristic entities. Unlike autoimmune diseases such as lupus, the psoriasis group exhibited global gene hypermethylation, with a strong correlation observed between individual patient PASI scores and 5-methylcytosine staining scores. However, MBD2 was notably downregulated in PBMCs from the disease group [82], and the mechanisms through which MBD2 might contribute to psoriasis development remain largely unexplored.

## Decoding MBD2-driven autoimmunity epigenetics for targeted therapy

Changes in gene expression programs are a fundamental aspect of autoimmune diseases. Increasing evidence indicates that alterations in epigenomic programming can stably and persistently modify gene function, thereby playing a critical role in autoimmune conditions. DNA methylation is recognized as a central epigenetic regulator, modulating cellular differentiation, disrupting immune homeostasis, and predisposing to autoimmune pathogenesis [83]. Pharmacological hypomethylation is linked to drug-induced lupus pathogenesis [84], as evidenced by genome-wide hypomethylation in SLE CD4^+^ T cells, particularly at interferon-regulated loci, establishing an epigenetic basis for autoimmune dysregulation [85]. Importantly, DNA methylation occurs not only within cells but also in target tissues. For instance, in multiple sclerosis, the promoter of PAD2(peptidyl arginine deiminase 2) is methylated at only one-third of the level found in normal tissue. This enzyme catalyzes the citrullination of myelin basic protein, leading to its accumulation in the white matter of the multiple sclerosis brain, which disrupts myelin stability and exacerbates the disease [86].

In the context of cancer therapy, the use of DNA methylation inhibitors and histone deacetylase (HDAC) inhibitors has demonstrated the potential to activate tumor-suppressor genes and halt tumor growth, as evidenced by the results of animal studies and selected clinical trials Nevertheless, it is evident that comprehensive disruption of epigenetic programming may result in both beneficial and detrimental alterations in gene expression. A notable example is DNA demethylation drugs, which inhibit tumor growth by activating tumor suppressor genes. However, they may also demethylate and activate pro-metastatic genes, thereby promoting tumor metastasis [87]. Consequently, the ability to target pivotal genes in specific cells or tissues is of paramount importance.

MBD2 is proposed to function as a 5-methylcytosine oxidase, converting 5mC to 5hmC while generating formaldehyde [88]. Global methylation analyses reveal no significant differences between Mbd2-deficient and wild-type mice, though restricted to CpG methylation at MspI/HpaII-sensitive loci in hepatic and splenic tissues [31]. Hypermethylation of several tumor-suppressor genes was observed in adenomas from APC^Min-/+^ MBD2^−/−^ mice with intestinal tumors. Recent studies also suggest that MBD2 may play a key role in DNA demethylation in lupus and other autoimmune disorders [89]. Significantly elevated MBD2 mRNA levels were observed in T cells from lupus patients, with a positive correlation between genomic hypermethylation and MBD2 mRNA levels [90]. Furthermore, evidence suggests that the demethylation of Th2 cytokines during T-cell maturation involves the non-coding region (CNS-1), which interacts with MBD2 in mature thymocytes [51]. This suggests that the protein may regulate the demethylation of this region. The aforementioned studies collectively highlight the potential of MBD2 to serve as a therapeutic target in diverse pathological conditions [34].

Furthermore, advancements have been made in the development of small-molecule inhibitors targeting the MBD2 gene, including MBD2 antisense oligonucleotide inhibitors. It has been demonstrated that these inhibitors lead to a reduction in MBD2 levels and the inhibition of tumor formation in human tumor xenografts in nude mice [91]. Further studies have demonstrated that MBD2 antisense oligonucleotide inhibitors enhance the methylation of pro-metastatic genes in breast and prostate cancer cells while simultaneously inhibiting cell invasion and metastasis. The MBD of MBD2 is central to its function, whereas the N-terminal region of MBD3 (such as the WIN motif in MBD3C) regulates gene expression by interacting with proteins like WDR5. This structural distinction theoretically provides a basis for selective inhibitor design; However, existing MBD2 inhibitors-KCC-07 primarily target the DNA-binding domain, raising concerns about cross-reactivity due to structural similarities between MBD2 and MBD3. To date, KCC-07 is the only MBD2 inhibitor explicitly reported to enhance cisplatin sensitivity in breast cancer models by inhibiting mitochondria-localized MBD2c [92]. Although this compound is proposed to exert therapeutic effects via targeting the MBD of MBD2, its subtype selectivity profile—particularly between MBD2a and MBD2c isoforms—remains insufficiently defined, and clinical translation has not been pursued due to unresolved pharmacological characterization. It would be of interest to investigate whether MBD2 inhibitors can reverse the demethylation of cytokines and other genes in CD4^+^ T cells from lupus patients [93]. If validated, MBD2 inhibition could offer a promising approach to treating the epigenetic defects associated with a range of autoimmune disorders. Moreover, the application of AI tools to develop small-molecule inhibitors targeting MBD2 [94], as well as other demethylases and demethylase-associated proteins, is crucial for advancing therapeutic strategies in autoimmune diseases.

## Discussion and summary

This article provides a comprehensive review of the role of MBD2 in immune cells and autoimmune disease mechanisms, with a focus on how MBD2 contributes to various autoimmune diseases through immune cells. We offer an in-depth analysis of the effects of MBD2 on both intrinsic and adaptive immune cells, linking these effects to autoimmune diseases such as SLE, rheumatoid arthritis, and urolithiasis, as well as exploring MBD2’s dual role in cancer progression and metastasis.

MBD2 also influences autoimmune diseases by regulating the development and differentiation of immune cells. Among adaptive immune cells, this review emphasizes how MBD2 precisely regulates T cell development and differentiation, thereby contributing to several autoimmune diseases, though studies on B cells remain relatively sparse. Research on intrinsic immune cells, such as DCs and macrophages, is similarly limited, and these areas urgently require further investigation. Furthermore, this review highlights MBD2’s crucial role in the progression of autoimmune diseases through the regulation of specific gene targets and cytokines. The autoimmune relevance of MBD2 splice variants (including MBD2a-MBD2c) and domain-specific functionalities of MBD2 have not been systematically mapped, creating barriers to developing precision epigenetic therapies. Therefore, future studies should explore the distinct effects of these splice variants on autoimmune diseases to advance the development of MBD2-targeted therapies. Future investigations should prioritize single-cell epigenomic profiling of MBD2-binding landscapes across immune cell subtypes to resolve context-dependent regulatory networks, coupled with the development of isoform-specific knockout models that delineate functional divergence between nuclear MBD2a and mitochondrial MBD2c variants. Concurrently, structure-guided drug design must focus on targeting IDR-mediated biomolecular condensates in autoimmune inflammation, as these integrated approaches will mechanistically validate MBD2’s pathogenic contributions while providing a roadmap for precision therapeutic development.
